# The Sigma-2 Receptor Selective Agonist Siramesine (Lu 28-179) Decreases Cocaine-Reinforced Pavlovian Learning and Alters Glutamatergic and Dopaminergic Input to the Striatum

**DOI:** 10.3389/fphar.2017.00714

**Published:** 2017-10-10

**Authors:** Anna M. Klawonn, Anna Nilsson, Carl F. Rådberg, Sarah H. Lindström, Mia Ericson, Björn Granseth, David Engblom, Michael Fritz

**Affiliations:** ^1^Cell Biology, Department of Clinical and Experimental Medicine, Linköping University, Linköping, Sweden; ^2^Addiction Biology Unit, Department of Psychiatry and Neurochemistry, Institute of Neuroscience and Physiology, University of Gothenburg, Gothenburg, Sweden

**Keywords:** addiction, cocaine, conditioned place preference, sigma-receptor, Siramesine, electrophysiology

## Abstract

Drug addiction is a chronic, debilitating disease that affects millions of people around the world causing a substantial societal burden. Despite decades of research efforts, treatment possibilities remain limited and relapse represents the most treatment-resistant element. Neurosteroid sigma-1 receptors have been meticulously studied in psychostimulant reinforced Pavlovian learning, while the sigma-2 receptor subtype has remained unexplored. Recent development of selective sigma-2 receptor ligands have now made it possible to investigate if the sigma-2 receptor system is a potential target to treat drug addiction. We examined the effect of the sigma-2 receptor agonist Siramesine (Lu 28-179) on cocaine-associated locomotion, Pavlovian learning, and reward neurocircuitry using electrophysiology recordings and *in vivo* microdialysis. We found that Siramesine significantly attenuated conditioned place preference acquisition and expression, as well as it completely blocked cocaine-primed reinstatement. Siramesine, in a similar manner as the selective sigma-1 receptor antagonist BD 1063, decreased acute locomotor responses to cocaine. Immunohistochemistry suggests co-expression of progesterone receptor membrane component 1/sigma-2 receptors and vesicular glutamate transporter 1 in presynaptic boutons of the nucleus accumbens (NAc). Whole-cell voltage clamp recordings of neurons in the NAc indicated that Siramesine decreases the presynaptic release probability of glutamate. Further, we demonstrated, via *in vivo* microdialysis, that Siramesine significantly decreased cocaine-evoked dopamine release in the striatum of freely moving mice. Collectively, these findings demonstrate that sigma-2 receptors regulate neurocircuitry responsible for positive reinforcement and thereby play a role in cocaine-reinforced Pavlovian behaviors.

## Introduction

Drug addiction is a chronic relapsing disorder with detrimental effects for the affected individuals and, despite decades of scientific effort, treatment possibilities remain scarce and relapse rates high (Volkow et al., 2016). Therefore, exploring new treatment targets against relapse is pivotal. Neurosteroid sigma-receptors (σRs) have previously been suggested to play a role in addiction pathology. Two subtypes of the σR have been identified based on drug selectivity and molecular mass: σ1R and σ2Rs (Cobos et al., 2008). Whereas the role of σ1Rs in addiction has been studied, very little is known about that of σ2Rs.

Cloning of the σ1R (Hanner et al., 1996) led to advances in the understanding of its function and linked it to modulation of various neurotransmitters (e.g., glutamate, GABA, serotonin, acetylcholine, etc.) and several neuropathologies (Cobos et al., 2008). In contrast, the identity of the σ2R remains controversial. A recent study identified the σ2R as the progesterone receptor membrane component 1 (PGRMC1) (Xu et al., 2011), and a follow-up study showed that the PGRMC1 protein levels correlate with the binding activity of a σ2R-selective probe (SW120) in the rat brain (Zeng et al., 2016). These findings, however, are debatable due to newer studies showing that PGRMC1 knockdown/out or knock-in interventions do not change σ2R binding of [3H]-DTG (1,3-di-o-tolylguanidine; a shared σ1R and σ2R ligand) in affinity assays (Abate et al., 2015; Chu et al., 2015). Nonetheless, compounds with selective binding affinities for σ2Rs relative to σ1Rs have been developed (Guo and Zhen, 2015). H. Lundbeck A/S generated the most selective ligand currently available: Lu 28-179/Siramesine. This is a highly selective σ2R agonist (σ2R Ki = 0.12 nM; σ1R 17 nM) (Perregaard et al., 1995) initially developed as an anxiolytic agent (Sánchez et al., 1997) and later found to have antidepressant-effects in rats (Sánchez and Papp, 2000). Furthermore, Siramesine has been used for labeling σ2R binding sites in rats, demonstrating the presence of σ2Rs in the nucleus accumbens (NAc), caudate putamen (CPu), and ventral tegmental area (VTA) (Søby et al., 2002). More recently, other σ2R ligands have been explored in cocaine addiction-related behaviors. 5-Bromo-*N*-[4-(6,7-dimethoxy-3,4-dihydro-1H-isoquinolin-2-yl)-butyl)]-2,3-dimethoxy-benzamide was found to antagonize cocaine-induced hyperlocomotion in mice (Lever et al., 2014) and Katz et al. (2016) observed effects of σ2R antagonists (CM398, CM353) on cocaine primed DTG self-administration. Finally, σ2R antagonist SN79 was shown to attenuate DTG-induced dopamine levels in the striatum (Garcés-Ramírez et al., 2011). Interestingly, the possible effects of Siramesine have not yet been explored in cocaine-reinforced behaviors.

In order to address the role of σ2Rs in behaviors relevant to psychostimulant addiction we tested Siramesine in acquisition, expression, and drug-primed reinstatement of cocaine-induced conditioned place preference (CPP), as well as in acute cocaine-induced hyperlocomotion using mice. Since Siramesine diminished some of the cocaine reinforced behaviors, we decided to employ a comparative pharmacological approach using the σ1R selective antagonist, BD 1063 (McCracken et al., 1999; Liu et al., 2005; Sabino et al., 2009). Furthermore, we elucidated how Siramesine affects glutamatergic input to cells in the NAc via whole-cell slice recordings and cocaine-evoked dopamine release within the striatum (NAc and CPu) via *in vivo* microdialysis in freely moving mice.

## Materials and Methods

### Animals

All animals used in this study were male C57/bl6 mice from Scanbur A/S. To reduce phenotypic variance (due to hierarchical structure, aggression, food availability, etc.) mice were single-housed at least 48 h before the behavioral experiments. The animals were housed with environmental enrichment (play tunnel and two types nesting material) and kept in a pathogen-free facility on a regular 12-h light/dark cycle. Food and water were provided *ad libitum*, and all experiments were performed during the light phase. All mice were 6 weeks or older at the start of the experiments, with exception of the animals used for electrophysiological recordings. Here brain slices were prepared from 4- to 6-week-old mice. All experiments involving the use of animals followed international and national guidelines (EU Directive 2010/63/EU for animal experiments) and were approved by the Research Animal Care and Use Committee in Linköping, Sweden.

### Drugs

Cocaine HCl was obtained commercially from Sigma-Aldrich and the Hässleholm Hospital Pharmacy, Sweden, and dissolved in saline. BD 1063 was purchased from Tocris and dissolved in saline. An initial batch of Siramesine was provided by H. Lundbeck A/S and a second batch was purchased commercially from Sigma-Aldrich. Siramesine was dissolved in saline using 1% Tween80 and subsequent sonication for 10 min.

### Locomotion

Locomotion was monitored in a standardized locomotor box [450 mm (W) × 450 mm (D) × 400 mm (H)] divided in four equal-sized compartments from Panlab, Harvard Apparatus. The locomotor activity of four mice was monitored simultaneously over 30 min using SMART VIDEO TRACKING software (Panlab, Harvard Apparatus). Total distance traveled (displayed in 5 min intervals) was used to evaluate the effects of BD 1063 or Siramesine under baseline conditions or acute cocaine-induced hyperlocomotion. On day 0, mice were injected intraperitoneally (i.p.) with either saline, BD 1063 (3 or 10 mg/kg) 10 min before the trial-start or Siramesine (0.3 or 1 mg/kg) 60 min prior to the trial. On day 1, mice received either BD 1063 (3 or 10 mg/kg) or Siramesine (0.3 or 1 mg/kg), respectively 10 or 60 min prior to an acute i.p. injection of cocaine (15 mg/kg). The specific time points for injection were chosen according to previous studies on behavioral effects of BD 1063 and Siramesine (Sánchez et al., 1997; McCracken et al., 1999).

### Place Conditioning Apparatus

We used a biased place conditioning procedure to measure preference by means of a three-chambered Panlab Spatial Place Preference Box (Harvard Apparatus). On day 1, during a 15-min pretest, the individual mouse was allowed to move freely between the chambers of the box. Time spent in each compartment was manually recorded during individual experiments done by three independent experimenters blinded to dose and treatment. To ensure explorative behavior during the pretest, each mouse had to cross the corridor, entering the opposing chamber a minimum of five times to be included in the experiment. Any animal that spent >66% of the pretest in either of the conditioning chambers was excluded from the study. In total *n* = 11 mice were excluded from the study due to these criterions.

Mice were assigned to vehicle- or cocaine-paired compartments in a manner to avoid reinforcing natural bias, i.e., cocaine injections were paired with the chamber that was least preferred during pretest. This method has been shown to produce reliable CPP responses to cocaine (Fritz et al., 2011). On day 2 in the morning, mice were injected with saline i.p. before confinement for 15 min in the vehicle chamber. Four hours later on the same day, the mice were trained to cocaine (15 mg/kg i.p.) in the opposite chamber. This alternating training procedure was continued for four consecutive days, until day 6, when the CPP was assessed during a 15-min posttest. The individual preference score was calculated by subtracting the time spent in the cocaine-paired chamber during the pretest from that of the posttest. The conditioning boxes were thoroughly cleaned after each mouse using warm water and a surface disinfectant.

In order to study reinstatement behavior, mice which expressed place preference toward cocaine underwent 6 days of extinction training. During extinction, the training sessions remained the same, except the mice were given saline (i.p.) in both the conditioning and vehicle chambers. Twenty-four hours after the last extinction training session, the mice underwent an extinction test for 15 min. Mice that lost >60% of their initial preference proceeded to the reinstatement session 24 h later. *n* = 11 mice failed to meet extinction criteria and were excluded from the study. To reinstate drug-seeking, the mice received 5 mg/kg cocaine i.p. immediately before a 15-min reinstatement test. The reinstatement score was calculated as time spent in the cocaine-associated chamber during the extinction test subtracted from that during the reinstatement test.

To assess whether σ1- or σ2Rs are involved in the acquisition, expression, and reinstatement of cocaine-induced place preference, mice were given an i.p. injection of BD 1063 (1, 3, or 10 mg/kg) 10 min prior to either cocaine training, CPP posttest, or drug-induced reinstatement test. Alternatively, Siramesine (0.1, 0.3, and 1 mg/kg) was administered i.p. 60 min before either cocaine training, CPP posttest, or drug-induced reinstatement test. To test if BD1063 or Siramesine had any inherently rewarding or aversive properties, an effective dose of each compound (10 or 0.3 mg/kg, respectively) was administered as the unconditioned stimulus according to the standard CPP protocol for four consecutive training sessions.

For natural reward place preference, we employed the same conditioning procedure as for cocaine. A small amount of Nutella (Ferrero on a gray tile was used as the unconditioned stimulus during training, compared to a clean gray tile in the non-conditioning chamber. On the day of the posttest, mice received i.p. injections of either saline, 10 mg/kg BD 1063 (10 min before the test), or 0.3 mg/kg Siramesine (60 min before the test).

### Food Intake

Before the experiments, mice were single-housed for at least 7 days in order to exclude any effects of hierarchical structure that would affect metabolism and food-intake. One hour before dark-period onset, food was withdrawn and saline or Siramesine (0.3 or 1 mg/kg i.p.) was injected, at lights out mice were given free access to a pre-weighed amount of standard chow. Food intake was measured 3, 6, and 12 h after refeeding.

### Electrophysiology

Coronal sections (250 μm) containing the NAc were prepared from male C57/bl6 mice. Mice were deeply anesthetized with isoflurane and decapitated before removal of the brain. Brains were quickly submerged in ice-cold artificial cerebral spinal fluid (aCSF) containing in mM: 124 NaCl, 26 NaHCO_3_, 3 KCl, 1.25 NaH_2_PO_4_, 3 myo-inositol, 2 MgCl_2_, 4 lactic acid, and 0.5 ascorbic acid. Slices were cut using a vibratome, in ice-cold aCSF with NaCl replaced by sucrose (in mM): 248 sucrose, 26 NaHCO_3_, 3 KCl, 1.25 NaH_2_PO_4_, 0.5 CaCl_2_, 6 MgCl_2_, 10 glucose, 3 myo-inositol, 0.5 ascorbic acid, and 4 lactic acid. To allow recovery, slices were incubated for a minimum of 90 min at 35°C in aCSF containing (in mM): 26 NaHCO_3_, 3 KCl, 124 NaCl, 1.25 NaH_2_PO_4_, 2 MgCl_2_, 4 CaCl_2_, 10 glucose, 3 myo-inositol, 0.5 ascorbic acid, and 4 lactic acid. Subsequently, individual slices were transferred to a recording chamber and perfused continuously (2 ml/min) with aCSF at 35°C containing (in mM): 26 NaHCO_3_, 3 KCl, 124 NaCl, 1.25 NaH_2_PO_4_, 2 MgCl_2_, 2 CaCl_2_, 10 glucose, 3 myo-inositol, 0.5 ascorbic acid, and 4 lactic acid. A 20 μM bicuculline (Tocris) was added to the aCSF to block GABA_A_-receptor mediated synaptic currents. All solutions were oxygenated with 95% O_2_/5% CO_2_. Siramesine (1.75 μM in 0.001% Tween 80) was bath applied to test the effect on spontaneous and evoked excitatory postsynaptic currents (EPSCs). Independent control experiments were conducted using 0.001% Tween 80, these demonstrated that the effects observed were due to Siramesine and not the solvent (data not shown). Glutamate receptor antagonists, DNQX and APV, were bath applied at concentrations of 20 μM. Recordings were started ca. 2 min after the drug was present in the recording chamber and lasted in average 10 min. Whole cell recordings were done with a cesium gluconate-based internal solution containing (in mM): 100 cesium gluconate, 10 NaCl, 10 HEPES, 20 TEA-Cl, 5 QX-314, 0.1 EGTA, and 1 MgATP. pH was adjusted to 7.3 and osmolality adjusted with H_2_O from hyperosmolar to 290 mOsm/l. Recording pipettes (4–6 MΩ) were pulled from borosilicate glass (World Precision Instruments). Neurons were visualized with a 40× water-immersion objective on an upright Axioskop FS microscope (Zeiss) equipped with infrared-differential interface contrast video microscopy via an Orca-R2 CCD camera (Hamamatsu). EPSCs were recorded in the whole-cell mode of the patch-clamp technique using an EPC9 amplifier (HEKA Elektronik, Lambrecht, Germany). Neurons were voltage clamped at -78 mV, adjusted for a liquid junction potential of 8 mV. In order to stimulate cortical input, a bipolar tungsten electrode was placed between the ventricle and anterior commissure bordering the NAc shell (Britt et al., 2012). Amplitude graded current pulses of 0.2 ms duration were produced by a STG4002 stimulus generator (Multichannel Systems). A master 8 pulse generator (AMPI) controlled the timing of voltage-pulses. Pulse-separation was 50 ms for paired pulse protocols. Stimulation intensities were adjusted to 2× the threshold-value. Data analysis was performed off-line using Igor Pro wavemetrics. Spontaneous EPSCs were recorded for 1 min. Events were analyzed offline using IgorPro with SpAcAn’s threshold-detection algorithm^1^ (Dugué et al., 2005).

### Immunohistochemistry

Brains were collected after intracardial perfusion with saline and 4% paraformaldehyde (PFA) in PBS. The brains were postfixed for 4 h in 4% PFA and subsequently cryoprotected in 30% sucrose PBS solution overnight. Coronal sections (40 μm) were cut on a freezing microtome, collected in cold cryoprotectant buffer (0.1 M phosphate buffer, 30% ethylene glycol, 20% glycerol), and stored at -20°C until further use. For immunofluorescent labeling, free-floating sections were washed in PBS, incubated in blocking solution (1% BSA and 0.3% Triton X-100 in PBS) and subsequently incubated with primary antibodies [Rabbit anti-PGRMC1, 1:200 (Sigma-Aldrich), guinea pig vesicular glutamate transporter 1 (VGLUT1), 1:500 (Synaptic Systems), mouse anti-tyrosine hydroxylase (TH), 1:1000 (Immunostar)] in blocking solution overnight. The following day, the sections were washed and incubated with secondary antibodies [Alexa Fluor 568 donkey anti-rabbit (1:200), Alexa Fluor 488 goat anti-guinea pig (1:500; Invitrogen), Alexa Flour 488 donkey anti-mouse (1:1000)] in blocking solution for 2 h. The sections were then washed and mounted on object glasses using Vectashield hardSet antifade mounting medium (Vector Laboratories). Sections were analyzed using a Zeiss Axio Observer Z1 fluorescence microscope connected to a Zeiss LSM 700 confocal unit with 405, 488, 555, and 639 nm diode lasers. Co-localization analysis of synaptic puncta was done by importing 63× confocal-images of the same frame-size (equivalent to the example in **Figure 5B**) into ImageJ and using the Plugin “Synapse Counter”^2^ (Dzyubenko et al., 2016).

### Stereotaxic Surgery

For all stereotaxic surgeries, mice were anesthetized with 5% isoflurane induction, placed in the stereotaxic frame (Leica Biosystems), and maintained at 1.0–1.5% isoflurane during the surgery. Chronic guide cannulas (AgnTho’s) for *in vivo* microdialysis probes were implanted using standard aseptic surgical and stereotaxic techniques. The guide cannula was fixed to the skull using two anchor screws and dental acrylic cement. A single stainless steel guide cannula with a dummy cannula was implanted at the injection site of the right hemisphere: striatal coordinates from bregma: AP, +1.1; ML, -1.2; and DV, -4.2. Dummy cannulas extending to the tip of the guide cannulas were removed after the stereotaxic surgery and replaced by microdialysis probes (membrane 1 × 0.6 mm, cut-off 100 kDa; AgnTho’s). The *in vivo* microdialysis experiments were performed after a 48-h recovery period. During the recovery period, mice were given analgesic (buprenorphine, 0.1 mg/kg i.p.). After the experiment, the mice were humanely euthanized by asphyxiation with CO_2_, and 1–2 μl methylene blue (Sigma-Aldrich) was injected through a microdialysis probe, without the membrane, to visually control for correct placement.

### *In Vivo* Microdialysis

Ringer solution was prepared from two stock solutions (refrigerated). Ringer stock A (10×) contained (in mM): 1470 NaCl, 30 KCl, 13 CaCl_2_, and 10 MgCl_2_ in 500 ml ultra-purified water. Ringer stock B (10×) consisted of 1 mM sodium phosphate buffer (NaH_2_PO_4_ × H_2_O) in 500 ml ultra-purified water. In the morning of each experiment, the final Ringer solution was made by mixing 3 ml Ringer stock A, 24 ml purified water, and 3 ml Ringer stock B (pH adjusted to 7.3). Mice were placed in a mouse infusion cage (CMA Microdialysis AB) and connected via FEP tubing (AgnTho’s) to a stainless steel dual swivel system (Harvard/Instech; CMA Microdialysis AB). A constant flow of 2 μl/min was guaranteed by a Univentor 801 Syringe pump (AgnTho’s). The dialysate was collected in FEP tubing connected to the outlet of the microdialysis probe. Every 10 min, the tubing was manually exchanged and the dialysate ejected by air pressure from a micropipette into polyethylene plastic vials (AgnTho’s), conjugated with 5 μl 50 mM HCl and immediately placed on ice. The collected samples were stored at -80°C until they were shipped on dry ice for analysis.

Mice received either a saline or a 0.3 mg/kg Siramesine injection i.p. and were left for 30 min in the infusion cage to ensure calibration of the microdialysis flow across the membrane. Subsequently, 3 × 10 min samples were collected for detecting baseline dopamine levels. Thereafter, both groups received a 15 mg/kg cocaine i.p. injection and another 7 × 10 min samples were taken.

### HPLC Analysis

Dopamine was separated and detected in the microdialysis samples using a high performance liquid chromatography coupled with electrochemical detection (HPLC-ED) system. This HPLC system consisted of a pump (Dionex P580, Kovalent AB, Västra Frölunda, Sweden), a stainless steel column (2 × 150 mm) packed with Nucleosil, 5 μm SA 100A (Phenomenex Skandinaviska Genetec, Västra Frölunda, Sweden) used at 32°C and an electrochemical detector (Decade, Kovalent AB) operated at 0.40 V versus the cell (Hy-REF). The mobile phase consisted of (in mM) 58 citric acid, 135 NaOH, 0.107 Na_2_-EDTA, and 20% methanol at a flow rate of 0.3 ml/min. The time of analysis from injection to injection was 6 min. An external standard containing 3.25 fmol/μl of dopamine was used to identify the dopamine peak.

### Statistics

Results are illustrated as mean ± SEM. Statistical comparison of the same cells in two different conditions during electrophysiology was done using Student’s paired *t*-test. When comparing more than two groups with comparable variances, one-way ANOVA or two-way ANOVA was used, followed by *post hoc* analysis with Dunnett’s (when comparing many groups to one control group) or Bonferroni’s (when comparing more than two groups to each other) multiple comparison tests to evaluate pairwise group differences. *p* < 0.05 was considered statistically significant. All statistical analyses were conducted using GraphPad Prism 6 software^3^.

## Results

### Locomotor Measurements

**FIGURE 1:**
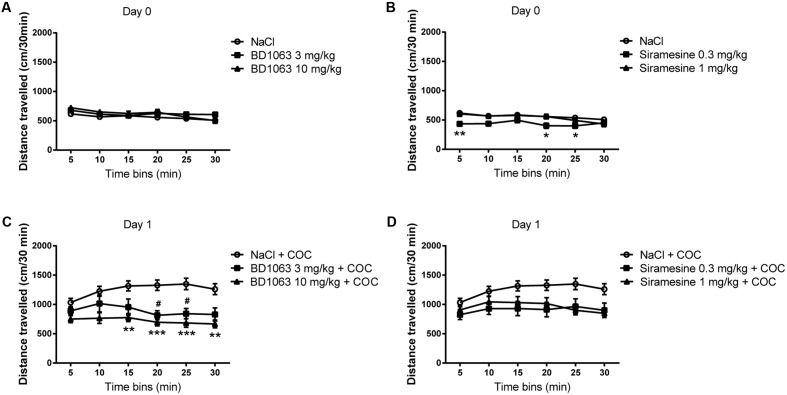
Locomotor Measurements. Mean ± SEM of baseline locomotor measurements during exposure to **(A)** NaCl (*n* = 24) vs BD 1063 [3 mg/kg (*n* = 8) or 10 mg/kg (*n* = 8)] and **(B)** NaCl (*n* = 24) vs Siramesine [0.3 mg/kg (*n* = 8) or 1 mg/kg (*n* = 8)]. BD 1063 had no effect on baseline locomotor activity, whereas Siramesine significantly attenuated it at 5, 20, and 25 min. **(C)** BD 1063 mitigates acute cocaine-induced hyperlocomotion in the last half of the 30-min tracking interval. **(D)** Siramesine also attenuated the acute cocaine-induced increase in locomotor activity as designated by significance in the two-way ANOVA. Locomotor measurements from NaCl-treated control mice were pooled and represented in both graphs. ^#,∗^*p* < 0.05, ^∗∗^*p* < 0.01, ^∗∗∗^*p* < 0.001. Two-way ANOVA followed by Bonferroni’s *post hoc* test.

To assess whether BD 1063, a σ1R selective antagonist, or Siramesine, a σ2R selective agonist, have an effect on baseline locomotor activity, mice were injected with saline or a high or a low dose of either compound. No differences in locomotor activity were found between the saline and the BD 1063 groups, **Figure 1A** [two-way ANOVA *F*(2,37) = 1.273, *p* > 0.05]. Whereas Siramesine at a dose of 0.3 mg/kg significantly decreased baseline locomotor activity after 5, 20, and 25 min [two-way ANOVA *F*(2,37) = 3.593, *p* < 0.05], **Figure 1B**. Next, we evaluated if BD 1063 or Siramesine were able to attenuate cocaine-induced hyper-locomotion. Mice received either saline, BD 1063 (3 or 10 mg/kg) or Siramesine (0.3 or 1 mg/kg) 10 or 60 min before an acute 15 mg/kg cocaine injection. Subsequently, the animals were monitored for 30 min. BD 1063 significantly decreased acute cocaine-induced hyper-locomotion during the last 15 min of the recorded behavior, **Figure 1C** [two-way ANOVA *F*(2,37) = 8.599, *p* < 0.01]. Treatment with Siramesine reduced the overall acute locomotor-effect of cocaine, **Figure 1D** [two-way ANOVA *F*(2,37) = 4.09, *p* < 0.05].

### Acquisition of Cocaine-Associated Place Preference

**FIGURE 2:**
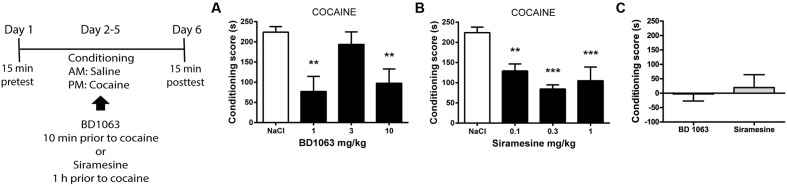
Acquisition of cocaine-associated place preference. Mean ± SEM of **(A)** the conditioning score for cocaine-induced place preference with or without BD 1063 [NaCl control (*n* = 10) vs 1 mg/kg (*n* = 5) vs 3 mg/kg (*n* = 6) vs 10 mg/kg (*n* = 6)]. **(B)** Siramesine attenuates the acquisition of cocaine-induced place preference [NaCl control (*n* = 10), 0.1 mg/kg (*n* = 6), 0.3 mg/kg (*n* = 6), 1 mg/kg (*n* = 5)]. CPP scores from NaCl-treated control mice were pooled and represented in both graphs. **(C)** Neither 10 mg/kg BD 1063 (*n* = 6) nor 0.3 mg/kg Siramesine (*n* = 6) cause an avoidance or approach behavior. ^∗∗^*p* < 0.01, ^∗∗∗^*p* < 0.001. One-way ANOVA followed by Dunnett’s *post hoc* test.

To elucidate if σ2Rs are involved in acquiring cocaine-associated Pavlovian learning in the same manner as σ1Rs, we used a place preference paradigm. Mice received saline or BD 1063 (1, 3, or 10 mg/kg) 10 min before each cocaine training session. As expected, 1 and 10 mg/kg BD 1063 significantly attenuated the acquisition of cocaine place preference, **Figure 2A** [one-way ANOVA *F*(3,23) = 7.245, *p* < 0.01]. In a second experiment, we administered Siramesine (0.1, 0.3, or 1 mg/kg) 60 min prior to the cocaine training sessions. All doses of Siramesine attenuated the acquisition of cocaine place preference, **Figure 2B** [one-way ANOVA *F*(3,23) = 13.66, *p* < 0.01]. To ensure that neither BD 1063 nor Siramesine were inherently rewarding or aversive, we used an effective dose of each compound (10 and 0.3 mg/kg, respectively) as an unconditioned stimulus. Neither substances caused avoidance or preference behavior, **Figure 2C**.

### Expression of Cocaine-Induced Place Preference

**FIGURE 3:**
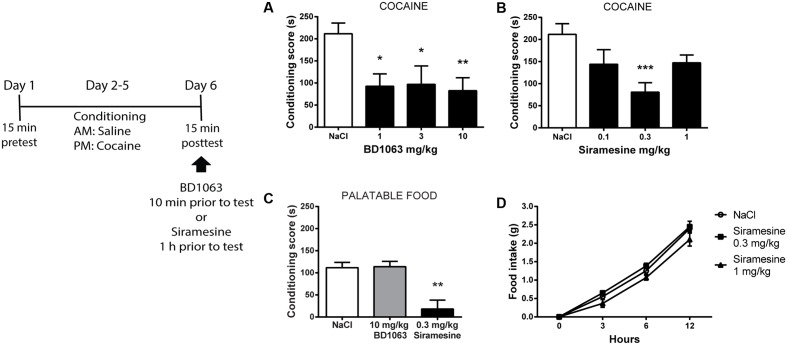
Expression of cocaine-induced place preference. Both, BD 1063 **(A)** (*n* = 12, 7, 9, and 13) and Siramesine **(B)** (*n* = 12, 8, 10, and 9) attenuate the expression of cocaine place preference as represented with mean ± SEM. CPP scores from NaCl-treated control mice were pooled and represented in both graphs. **(C)** Siramesine, but not BD 1063, reduces the expression of palatable food place preference (*n* = 5, 5, and 5). **(D)** Siramesine has no effect on cumulative food intake (*n* = 6, 6, and 6). ^∗^*p* < 0.05, ^∗∗^*p* < 0.01, ^∗∗∗^*p* < 0.001. One-way ANOVA followed by Dunnett’s *post hoc* test.

We next investigated if σ2Rs play a role similar to σ1Rs during expression of learned cocaine associations. To do so, mice that had undergone cocaine-conditioning, were given either saline or BD 1063 (1, 3, or 10 mg/kg) 10 min prior to the place preference test. All three doses of BD 1063 significantly lowered the expression of cocaine-place preference, **Figure 3A** [one-way ANOVA, *F*(3,37) = 4.247, *p* < 0.05]. In a subsequent experiment, conditioned mice were administered either saline or Siramesine (0.1, 0.3, or 1 mg/kg) 60 min before the posttest. Siramesine was also able to significantly reduce the expression of cocaine-induced place preference at a dose of 0.3 mg/kg, **Figure 3B** [one-way ANOVA, *F*(3,35) = 5.374, *p* < 0.01]. Subsequently, we examined if the effects of BD 1063 and Siramesine were specific to cocaine-reinforced motivational learning or affect natural reward learning as well. Whereas BD 1063 did not alter Nutella-induced place preference, Siramesine significantly attenuated the expression of place preference to this palatable food, **Figure 3C** [one-way ANOVA, *F*(2,12) = 12.66, *p* < 0.01]. To assess, if this effect was due to metabolic changes, we monitored regular food intake for 12 h in animals injected with either saline or Siramesine (0.3 or 1 mg/kg). No effect of Siramesine on food-intake was found, **Figure 3D** [two-way ANOVA, *F*(2,45) = 3.567, *p* > 0.05].

### Drug-Primed Reinstatement of Cocaine Place Preference

**FIGURE 4:**
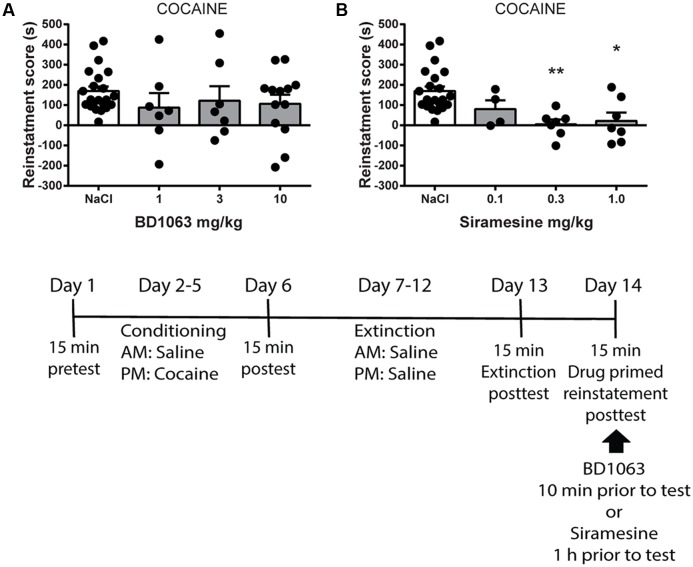
Drug-primed reinstatement of cocaine-place preference. **(A)** BD 1063 does not prevent drug-primed reinstatement (*n* = 22, 7, 7, and 13) as shown by mean ± SEM. In contrast, **(B)** Siramesine blocks cocaine-primed reinstatement of a previously extinguished place preference (*n* = 22, 4, 7, and 7). CPP scores from NaCl-treated control mice were pooled and represented in both graphs. ^∗^*p* < 0.05, ^∗∗^*p* < 0.01. One-way ANOVA followed by Dunnett’s *post hoc* test.

Finally, we tested if BD 1063 or Siramesine are capable of preventing drug-primed reinstatement of cocaine-induced place preference. Mice were trained to express cocaine-CPP and subsequently underwent an extinction protocol. Animals that displayed significant loss of preference were submitted to reinstatement the following day. Mice received saline, BD 1063 (1, 3, or 10 mg/kg) or Siramesine (0.1, 0.3, or 1 mg/kg), 10 or 60 min, respectively, prior to reinstatement using a low dose of cocaine (5 mg/kg). The mice were then immediately tested for re-expression of cocaine-seeking behavior. BD 1063 was ineffective at attenuating cocaine-primed reinstatement, **Figure 4A** [one-way ANOVA, *F*(3,45) = 0.8225, *p* > 0.05]. In contrast, drug-primed reinstatement of cocaine seeking was abolished in mice that had received at least 0.3 mg/kg of Siramesine, **Figure 4B** [one-way ANOVA, *F*(3,36) = 7.261, *p* < 0.01].

### Whole-Cell Patch Clamp Recordings of Nucleus Accumbens Neurons

**FIGURE 5:**
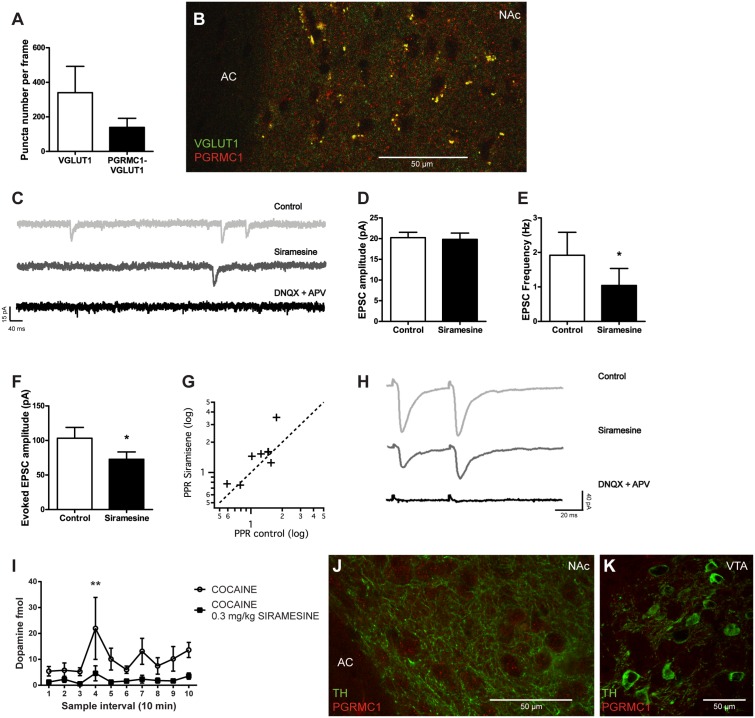
Effects of Siramesine on glutamate release probability in the nucleus accumbens and dopamine input to the striatum. Mean ± SEM of **(A)** numbers of VGLUT1 expressing boutons vs VGLUT1 boutons co-localized with PGRMC1/σ2R puncta in the NAc (*n* = 4). **(B)** A 63× confocal image showing co-localization of VGLUT1 (green) and PGRMC1/σ2R (red) in terminals adjacent to cell somas of NAc near the anterior commissure (AC). **(C)** Spontaneous EPSCs before and after bath-application of drugs. Siramesine decreases the frequency of spontaneous events, while DNQX and APV abolishes them completely. **(D)** Siramesine does not affect the amplitude of spontaneous EPSCs **(E)** but significantly decreases their frequency as represented in mean ± SEM (*n* = 8). **(F)** Siramesine decreases the amplitude of the first evoked EPSCs (*n* = 8), **(G)** and increases the paired pulse ratio (PPR) in six out of eight cells. **(H)** Example traces from the paired pulse stimulus, showing how Siramesine reduces EPSC amplitude and increases PPR. Evoked EPSCs are abolished by DNQX and APV. **(I)** Dopamine concentrations in the striatum (NAc and CPu) prior to and following cocaine administration. When Siramesine was injected 30 min prior to sampling, it significantly decreased cocaine-induced dopamine levels in the striatum. Cocaine was i.p. injected at the third sample-interval (NaCl *n* = 8, Siramesine *n* = 9). **(J)** A 63× confocal image of TH-positive fibers (green) and PGRMC1/ σ2R (red) in terminals adjacent to cell somas of NAc near the anterior commissure (AC). **(K)** A 63× confocal image of VTA TH-positive neurons (green) without any PGRMC1/σ2R (red) co-labeling of immunoreactivity. ^∗^*p* < 0.05, ^∗∗^*p* < 0.01. Student’s paired *t*-test or two-way ANOVA followed by Bonferroni’s *post hoc* test.

Medial prefrontal cortex (mPFC) glutamatergic synaptic transmission to NAc neurons is necessary for reinstatement of drug seeking (LaLumiere and Kalivas, 2008) and has been recognized as a primary target for cocaine (Suska et al., 2012). σ2R agonism has previously been shown to inhibit voltage-gated calcium channels, including the P- and N-types that are important for triggering transmitter release, indicating that σ2Rs could modulate the efficiency of synaptic vesicle release (Zhang and Cuevas, 2002). To determine if the putative σ2R (PGRMC1) is present on glutamatergic terminals in the NAc, we performed fluorescence immunohistochemistry. Presynaptic terminals of glutamatergic neurons were identified by VGLUT1 immunoreactivity. PGRMC1-labeling was found at approximately 40% of such synapses in the NAc, as illustrated by co-localization between PGRMC1 and VGLUT1 immunoreactivity, **Figures 5A,B**. It should be noted that despite the eminent expression of PGRMC1 on VGLUT1-positive terminals, several PGRMC1 puncta in the NAc were VGLUT1-negative. To investigate if σ2Rs have an effect on synaptic vesicular release, we performed whole-cell voltage-clamp recordings of NAc neurons. Siramesine significantly reduced the frequency (Student’s paired *t*-test, *p* < 0.05) of spontaneous excitatory synaptic events (sEPSCs), without affecting their amplitude, **Figures 5C–E**. The glutamatergic identity of these events was confirmed at the end of recording sessions by glutamate receptor antagonists DNQX and APV, **Figure 5C**. An acute decrease in sEPSC frequency is commonly interpreted as a decrease in transmitter release probability from the presynaptic bouton.

To further investigate the effect of Siramesine on transmitter release, cortico-striatal fibers were stimulated with a tungsten electrode. Evoked EPSCs recorded in NAc neurons were significantly smaller after application of the σ2R agonist, **Figures 5F,H** (Student’s paired *t*-test, *p* < 0.05). Further, during paired pulse stimulation (50 ms separation), the paired pulse ratio (EPSC_2_/EPSC_1_) was increased in six out of eight cells, **Figures 5G,H**. These findings support that the activation of σ2Rs indeed reduces the efficiency by which synaptic vesicles are released at cortico-striatal synapses in the NAc. Hence, Siramesine could mediate its effect on positive reinforcement behavior by influencing the release-probability of neurotransmitters signaling reward.

### *In Vivo* Microdialysis

To further investigate the role of σ2Rs in the reward neurocircuitry, we examined if Siramesine affects dopamine levels in the striatum using *in vivo* microdialysis. Mice were given either saline or 0.3 mg/kg Siramesine and were subsequently attached to the microdialysis tubing and placed in the infusion cage. After an initial 30 min period, which was used to stabilize flow and neurotransmitter exchange, three 10 min baseline samples were taken. Immediately after the third sample was taken, all mice received a 15 mg/kg cocaine injection and another seven samples were collected. Two-way ANOVA [*F*(9,150) = 1.723; *p* = 0.08 group-factor; *F*(1,150) = 26.11 *p* < 0.01 sample-factor] and *post hoc* analysis (Bonferroni multiple comparison; *p* < 0.01 for sample interval 4) revealed that Siramesine attenuated cocaine-evoked dopamine release in the striatum, **Figure 5I**. To determine if the protein PGRMC1 (the putative σ2R) is present on dopaminergic terminals in the striatum or on dopamine neurons in the VTA, we performed fluorescence immunohistochemistry targeting TH, the rate-limiting enzyme in catecholamine synthesis. We found no expression of PGRMC1 on TH-positive terminals in the striatum (example shown from NAc), **Figure 5J**, nor on TH-positive neurons in the VTA, **Figure 5K**.

## Discussion

In this study, we used a preclinical model of Pavlovian reinforcement learning to demonstrate the potential of utilizing σ2Rs as treatment targets against psychostimulant drug addiction. The selective σ2R agonist Siramesine significantly attenuated acquisition, expression, and drug-primed reinstatement of cocaine-induced place preference. Siramesine did this most effectively at a dose of 0.3 mg/kg. It did so in a manner similar to the selective σ1R antagonists BD 1063 and previously reported BD 1047 (Maurice et al., 2002; Maurice and Romieu, 2004), with the exception that it proved to be more effective against drug-primed reinstatement than BD 1063. The two receptor subtypes also differed with respect to baseline locomotor activity, whereas σ1R antagonism had no effect, Siramesine significantly decreased locomotor movement. In our study and in others, BD 1063 robustly and reproducibly attenuated cocaine-induced hyperlocomotion (McCracken et al., 1999; Matsumoto et al., 2001), the same was observed for Siramesine. This is in accordance with recently published findings on a selective σ2R ligand (Lever et al., 2014).

We cannot exclude that the dissimilarities in effects between BD 1063 and Siramesine could be due to diverse pharmacokinetic properties, as the drugs were administered at different time-points and the specific pharmacokinetic profile of BD 1063 has not yet been fully characterized. But since we see effects of BD 1063 on cocaine-induced locomotion, acquisition, and expression of CPP, it seems unlikely that the compound would not be functionally active within this timeframe. Furthermore, similar studies on drugs of abuse-induced behaviors have reported effects after pre-treatment with BD 1063 within the same time-frame as our experiments (McCracken et al., 1999; Sabino et al., 2009). Hence, from our results, it seems likely that Siramesine possess certain different capabilities than BD1063 on natural (baseline locomotion and palatable food preference) and cocaine-related (drug-primed reinstatement) behaviors. This suggests that Siramesine, on the contrary to BD1063, may have a treatment potential against cocaine relapse.

A peculiar phenomenon of Siramesine is the absence of a clear dose–response relationship, as both high and low doses produced weaker effects in acquisition, expression, and reinstatement of CPP. This is not an uncommon occurrence for the σ-receptor system and has previously been reported in studies using σ1R agonists (review of literature, Cobos et al., 2008). The U-shaped dose–response relationship is explained by the biological process of hormesis. In the case of σ1R agonism, hormesis is assumed to occur as a consequence of the modulatory function these receptors exert over various neurotransmitter-systems (Cobos et al., 2008). Our findings indicate that similar mechanisms could be present during σ2R agonism. This is supported by Sánchez and Papp (2000), who found a comparable dose–response curve for Siramesine on depression-like phenotype. More specifically, σ1R hormesis has been proposed to occur as a consequence of postsynaptic adaptations via glutamatergic NMDA-receptors, but it could also be related to a presynaptic mechanism. Our findings would suggest the latter for the σ2R, since we see an effect on release-probability of glutamate.

Our results show that σ2Rs provide negative control over glutamate release from cortical structures in the NAc. It has been demonstrated that glutamatergic input to the NAc, including afferents from mPFC, drives reward seeking (Britt et al., 2012). The Siramesine-induced reduction in release probability at glutamatergic synapses with NAc neurons may explain the decrease in motivational drive toward cocaine and palatable food, which was observed in our study. Changes in excitatory input to medium spiny neurons (MSNs) of the NAc could elucidate the observed attenuation in cocaine evoked dopamine-release. It has previously been shown that a significant number of MSNs, projecting to the VTA, target GABAergic interneurons and thereby regulate dopamine tone (Xia et al., 2011). A decrease in excitability of such MSNs, due to Siramesine acting on glutamatergic terminals, could result in lowered dopamine release.

Furthermore, despite that PGRMC1 is widely expressed throughout the brain as shown with *in situ* hybridization (Allen Brain Atlas^4^), we observed no co-labeling with TH-positive neurons in the VTA nor on TH-positive terminals in the striatum. If the σ2R indeed is the same protein as PGRMC1, it is unlikely that the decrease in cocaine evoked dopamine levels is due to a direct effect of Siramesine on VTA dopamine neurons. Finally, we noticed the presence of a number of PGRMC1-positive puncta in the NAc that are VGLUT1- and TH-negative. These could as well contribute to the observed effects on behavior. Irrespectively, it is clear that Siramesine has significant effects on classical motivational neurocircuitry.

It is interesting to note, that whereas previous studies have shown a role for σ1R antagonism in treating drug addiction (McCracken et al., 1999; Maurice et al., 2002; Maurice and Romieu, 2004; Romieu et al., 2004; Sabino et al., 2009; Fritz et al., 2011), the effects we observed are due to σ2R agonism. This would indicate that the two specific σR-subtypes have dissimilar molecular responses to agonists and antagonists, or alternatively execute their function via opposing neurocircuitry. Some evidence support the first alternative. We found that σ2Rs decrease glutamate release in NAc. In contrast, it has been reported that σ1Rs enhance glutamate release in various structures (i.e., hippocampus and cortex) (Meyer et al., 2002; Dong et al., 2006). Similarly, our results showed σ2R agonism decreased dopamine release in the striatum, while a study using the selective σ1R agonist, PRE-084, demonstrated significantly increased dopamine levels (Garcés-Ramírez et al., 2011).

Yet, some dichotomies presented in the current literature on σ2Rs remain to be solved. A recent study (Katz et al., 2016) showed that cocaine primed self-administration of DTG (a combined σ1R and σ2R agonist) can be attenuated in a dose-dependent manner using σ2R selective antagonists (CM398 and CM353). This would indicate that σ2R agonism has positive reinforcing properties, which was not observed in our study. Further enquiries are needed to provide a better understanding of the identity and molecular mechanisms of σ2R signaling. Also, future preclinical investigations of Siramesine would be of importance in order to assess how it influences operant self-administration in rodents.

Finally, since Siramesine already has been tested in a clinical-phase II trial for anxiety, it would be relevant to expand clinical trials to test its efficacy in preventing relapse in patients being treated for cocaine addiction.

## Author Contributions

AK and MF were responsible for the overall study design. AK, CR, and MF performed conditioning experiments and AK did the locomotion trials. AN conducted the food-intake experiment. AK did the electrophysiological recordings under the supervision of SL and with methodological inputs from BG. Histological analysis was done by AK. Stereotaxic surgeries and *in vivo* microdialysis sampling were conducted by AK and MF. ME provided HPLC analysis of the *in vivo* microdialysis samples. MF and AK wrote the manuscript, with input from DE. All authors discussed the results and commented on the manuscript.

## Conflict of Interest Statement

The authors declare that the research was conducted in the absence of any commercial or financial relationships that could be construed as a potential conflict of interest.

## Footnotes

1 http://www.spacan.net/

2 https://github.com/SynPuCo/SynapseCounter

3 www.graphpad.com

4 http://mouse.brain-map.org/gene/show/32808
